# Supplementary material for: Posterior Lissencephaly Associated with Subcortical Band Heterotopia Due to a Variation in the CEP85L Gene: A Case Report and Refining of the Phenotypic Spectrum
Source: Genes (Basel). 2021 Aug 5;12(8):1208. doi: 10.3390/genes12081208 (PMC8391275; doi:10.3390/genes12081208)
Supplement: Supplementary file 1 [file genes-12-01208-s001.zip › GENES_Contro_CEP85L_Figure S1 Tables S1 S2 S4.pdf]

Figure S1 - Analysis of the *CEP85L* transcript in patient 1. (A) Graphical representation of *CEP85L* exons 1-3 (not drawn to scale); the position of the primers used on cDNA is indicated by red arrows. (B) cDNA amplification of exon 1-3 region in cDNA from two wild-type controls (proband's father, f, and mother, m) and the proband, p; nt=no-template control, mw=molecular weight marker (Gel Pilot 100 bp ladder, QIAGEN, Maryland, USA). The proband's lane presents both the expected 424 bp amplicon and a band at lower molecular weight. (C) Sequencing of the cDNA from the proband's low-weight band demonstrates the formation of a 1/3 exon junction.

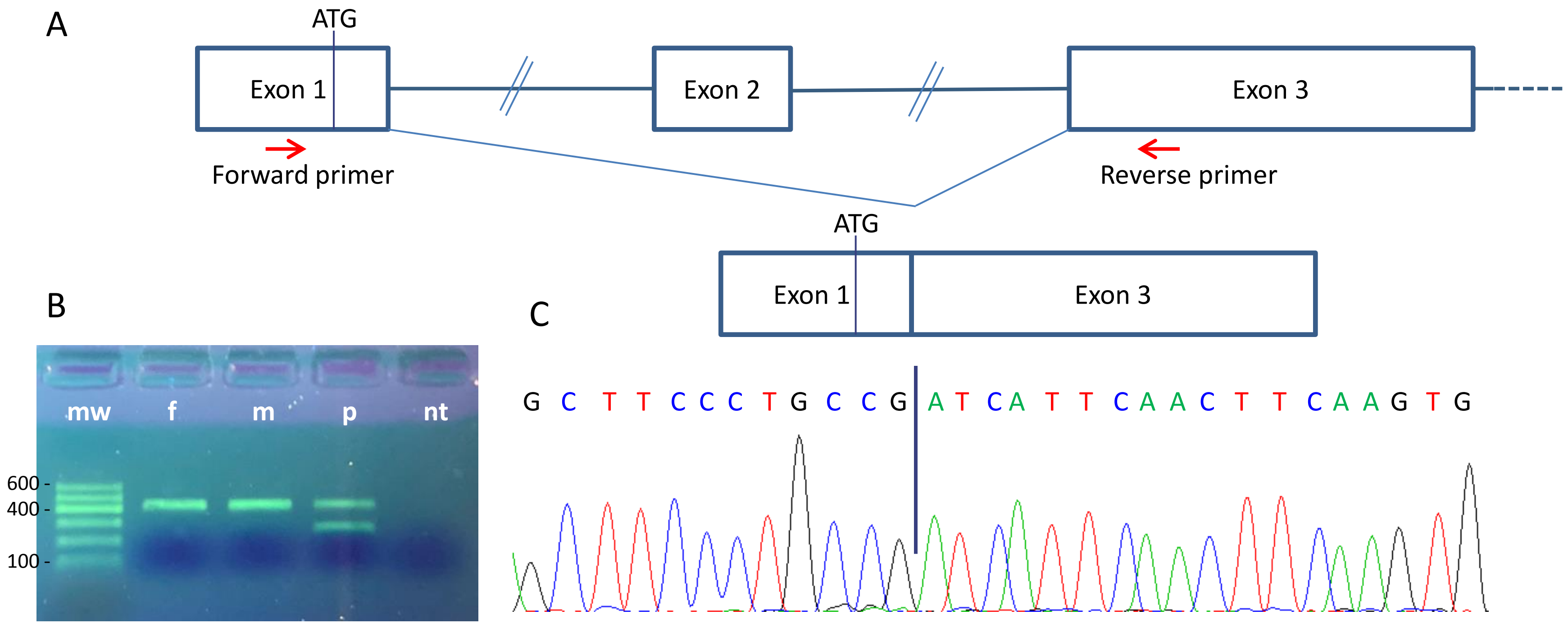

Table S1 - Sequence (5’ -> 3’) of the primers used for analysis of the *CEP85L* cDNA (and β-actin control)

CEP85L_cDNA_1F	AAGAAGCAGGAGGACGGAC
CEP85L_cDNA_1R	TGGTGTCAATGATTCCCTAAG
ACTB_F	GCAAGAGATGGCCACGGCTG
ACTB_R	ATCCACACGGAGTACTTGCG

Table S2. List of the 182 genes associated with brain malformations analyzed throught a targeted Next Generation Sequencing (NGS) custom panel.

ACTB	*CELSR2*	*EIF4EBP1*	*KIF5C*	*PAX6*	*RPS6*	*TMEM237*
ACTG1	*CENPE*	*EML1*	*KIF7*	*PCLO*	*RPS6KB1*	*TMEM67*
ADGRG1	*CENPJ*	*EMX2*	*KNL1*	*PDE6D*	*RPTOR*	*TMTC3*
AHI1	*CEP135*	*ERMARD*	*LAMA2*	*PHC1*	*RTTN*	*TSC1*
AKT1	*CEP152*	*EXOSC3*	*LAMB1*	*PI4KA*	*RXYLT1*	*TSC2*
AKT1S1	*CEP290*	*EXOSC8*	*LAMC3*	*PIK3CA*	*SASS6*	*TSEN15*
AKT3	*CEP41*	*EZH2*	*LARGE1*	*PIK3R2*	*SEPSECS*	*TSEN2*
AMPD2	*CEP63*	*FAT4*	*MCPH1*	*PLK4*	*SHH*	*TSEN34*
ANKLE2	*CHMP1A*	*FIG4*	*MFSD2A*	*POGZ*	*SIX3*	*TSEN54*
ARFGEF2	*CIT*	*FKRP*	*MKS1*	*POMGNT1*	*SLC25A19*	*TUBA1A*
ARL13B	*CLP1*	*FKTN*	*MLST8*	*POMGNT2*	*SRGAP2*	*TUBA8*
ARX	*CNTNAP2*	*FLNA*	*MTOR*	*POMT1*	*STAMBP*	*TUBB*
ASNS	*COL4A1*	*FOXC1*	*MYCN*	*POMT2*	*STIL*	*TUBB2A*
ASPM	*COL4A2*	*GLI2*	*NDE1*	*PPP1R15B*	*STRADA*	*TUBB2B*
ATRX	*CRADD*	*GMPPB*	*NEDD4L*	*PQBP1*	*STX7*	*TUBB3*
B3GALNT2	*CSPP1*	*GNAQ*	*NFIX*	*PTCH1*	*TBC1D20*	*TUBG1*
B4GAT1	*CTNNB1*	*GPSM2*	*NHEJ1*	*RAB18*	*TBC1D23*	*TUBG2*
B9D1	*CUL4B*	*IER3IP1*	*NPHP1*	*RAB3GAP1*	*TBC1D7*	*VLDLR*
C5orf42	*DAG1*	*INPPSE*	*NPRL2*	*RAB3GAP2*	*TBCD*	*VPS53*
CC2D1A	*DCHS1*	*ISPD*	*NPRL3*	*RALGAPA1*	*TCTN1*	*VRK1*
CC2D2A	*DCX*	*KANSL1*	*NSD1*	*RALGAPA2*	*TCTN2*	*WDR62*
CCND2	*DEPDC5*	*KAT6A*	*OCLN*	*RALGAPB*	*TCTN3*	*WDR81*
CDK5	*DEPTOR*	*KATNB1*	*OFD1*	*RARS2*	*TGIF1*	*YWHAE*
CDK5RAP2	*DNAI1*	*KIAA0556*	*OPHN1*	*RELN*	*TMEM138*	*ZIC1*
CDK6	*DYNC1H1*	*KIAA0586*	*ORC1*	*RICTOR*	*TMEM216*	*ZNF335*
CDON	*EIF4E*	*KIF11*	*PAFAH1B1*	*RPGRIP1L*	*TMEM231*	*ZNF423*

Table S4 - *In silico* predictions on how the different variants at CEP85L exon 2 donor site affect the canonical 5’ splice site

**NM_001042475.3:**	**MaxEnt (score)**	**NNSPLICE (confidence)**	**NetGene2 (confidence)**	**SPiCE (probability)**	**Patient #**[†]
WT	9.60	0.89	0.79	0.02686	
c.232+1del	-9.38	0.00	0.00	1	1
c.232+1G>T	1.09	0.00	0.00	1	10
c.232+3G>T	5.61	0.46	0.00	0.89191	11, 12
c.232+5G>T	4.41	0.00	0.00	0.99519	20, 21
c.232+5G>A	5.54	0.00	0.00	0.98584	13, 14

†Case numbers refer to patients as presented in Table S3
